# Meta-analysis of melatonin treatment and porcine somatic cell nuclear transfer embryo development

**DOI:** 10.1590/1984-3143-AR2021-0031

**Published:** 2021-11-04

**Authors:** Zhenhua Guo, Wengui Chen, Lei Lv, Di Liu

**Affiliations:** 1 Key Laboratory of Combining Farming and Animal Husbandry, Heilongjiang Academy of Agricultural Sciences, Animal Husbandry Research Institute, Ministry of Agriculture and Rural Affairs, Harbin, P. R., China; 2 Animal Science and Technology College, Northeast Agricultural University, Harbin, P. R., China; 3 Wood Science Research Institute of Heilongjiang Academy of Forestry, Harbin, P. R., China

**Keywords:** meta-analysis, melatonin, SCNT, porcine

## Abstract

Porcine somatic cell nuclear transfer (SCNT) plays an important role in many areas of research. However, the low efficiency of SCNT in porcine embryos limits its applications. Porcine embryos contain high concentrations of lipid, which makes them vulnerable to oxidative stress. Some studies have used melatonin to reduce reactive oxygen species damage. At present there are many reports concerning the effect of exogenous melatonin on porcine SCNT. Some studies suggest that the addition of melatonin can increase the number of blastocyst cells, while others indicate that melatonin can reduce the number of blastocyst cells. Therefore, a meta-analysis was carried out to resolve the contradiction. In this study, a total of 63 articles from the past 30 years were analyzed, and six papers were finally selected. Through the analysis, it was found that the blastocyst rate was increased by adding exogenous melatonin. Melatonin had no effect on cleavage rate or the number of blastocyst cells, but did decrease the number of apoptotic cells. This result is crucial for future research on embryo implantation.

Porcine somatic cell nuclear transfer (SCNT) plays an important role in many areas of scientific research. First of all, pigs can be one potential choice of candidates for human organ transplantation after gene modification in the future, since organs with low rejection rates can be obtained through SCNT after gene editing (Rao et al., 2016). Second, porcine SCNT technology is also used to study the swine totipotency of cells (Xu et al., 2020; Zhou et al., 2020). In swine embryo early development, the totipotency of cells is limited to the blastomeres. With further development of embryos, totipotency is gradually lost, and cells are differentiated into different types of pluripotent stem cells. Third, porcine SCNT can be used to establish disease models (Xie et al., 2018). Fourth, it is a good sample in research on nuclear reprogramming after SCNT (Sper et al., 2017). However, the efficiency of SCNT in porcine animals is low, like other mammalian SCNT (Song et al., 2014). Specially, in the embryo transfer step, 200–300 SCNT embryo need to be transferred in one surrogate sow (Huang et al., 2014). The application of SCNT is thus limited.

Porcine embryos contain high concentrations of lipids that provide energy for early embryonic development. However, this biological characteristic also makes the embryo vulnerable to the harmful effects of oxidative stress (Sturmey et al., 2009). In the process of *in vitro* culture, embryos face higher oxidative stress than embryos *in vivo*. *In vivo* embryos produce less reactive oxygen species (ROS) (Choi et al., 2008). With *in vitro* culture, there will be excessive ROS that react with lipids, leading to the destruction of cell membrane integrity, changes in the structure and function of proteins, and damage to nucleic acids (Tamura et al., 2012). Reducing ROS can improve embryonic development (Khalil et al., 2013). Thus, melatonin has been used to reduce ROS damage in SCNT embryos (Qu et al., 2020a). There are many reports concerning the effect of exogenous melatonin on SCNT in porcine species (Nakano et al., 2012; Liang et al., 2017), Some studies suggest that melatonin can increase the number of blastocyst cells (Liang et al., 2017; Qu et al., 2020a), while other studies have suggested that the number of cells has been reduced (Nakano et al., 2012). Therefore, we conducted a meta-analysis to resolve this contradiction.

## Methods

### Database search strategy and data extraction

**Figure 1:**
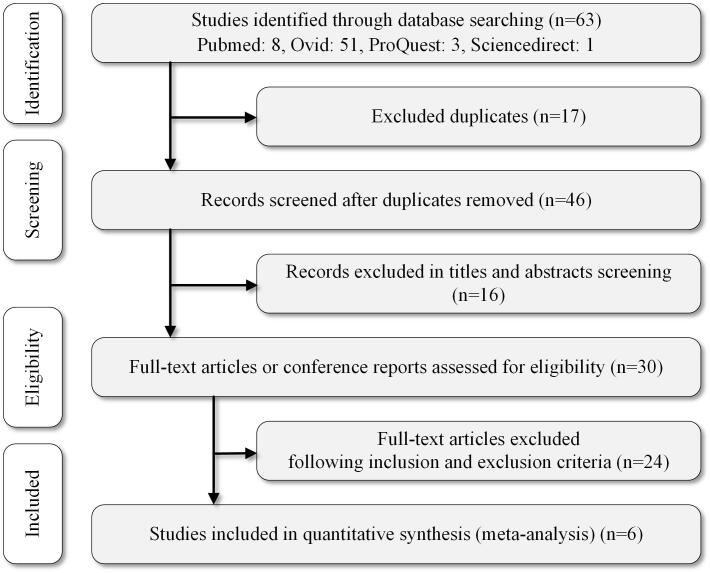
Summary of study selection.

A search was conducted using PubMed, Ovid, ScienceDirect, and ProQuest for studies from January 1, 1990 to September 2020. In all, 63 articles were found with the keywords porcine and (SCNT or clone or cloned) and melatonin. The first two authors searched independently (Table 1, Figure 1). In case of disputes, the third author decided to include or exclude the articles. In this step, statement of the blastocyst rate was a necessary condition. Only those articles that mentioned melatonin treatment in combination with a control group were included.

**Table 1:** Inclusion and exclusion criteria.

**Inclusion**	**Exclusion**
Species evaluated included, but were not limited to, porcine animals	Porcine animals were not used
English literature	Non-English
Melatonin treatment alone or with other treatment of porcine animals	No melatonin treatment of porcine animals
SCNT data included	No SCNT data

### Data analysis

The data for blastocyst rate, cleavage rate, blastocyst cell number, and apoptotic cell number in each paper were extracted and counted one by one. The blastocyst rate and cleavage rate were processed as dichotomous variables. The number of blastocyst cells and apoptotic cells were considered as continuous variables. Those studies with heterogeneity were assumed as random effects models. A fixed effect model was assumed for studies without heterogeneity. Data analyses were conducted with Review Manager (Version 5.4, Copenhagen: Nordic Cochrane Centre, Cochrane Collaboration). Publication bias was observed by using a funnel plot. If the data were evenly distributed, this indicated no bias.

## Results

**Figure 2:**
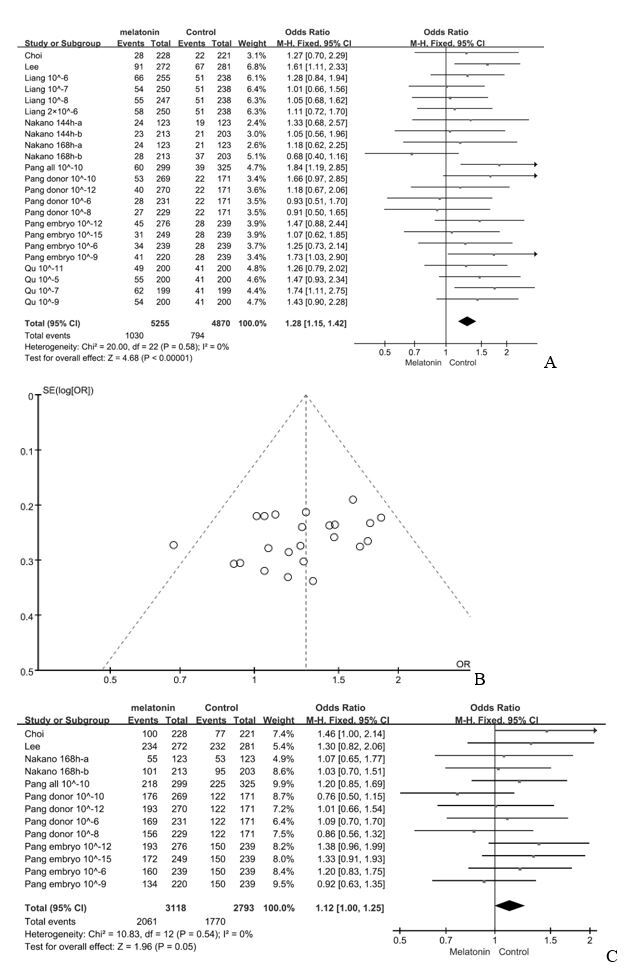
Forest plot of melatonin treatment effects on (A) porcine blastocyst rate and (B) cleavage rate, CI = 95%. (C) funnel plot of melatonin treatment effects on porcine blastocyst rate. “Liang 10 ^ - 6” indicates that the additive amount is 10 ^ - 6 in Liang's study; “Nakano 144h-a” refers to group A with 144-hour culture time in Nakano's study; “Pang donor 10 ^ - 6” refers to the treatment of the donor in Pang's study with an additive amount of 10 ^ - 6; and “all” refers to the treatment of donor and embryo in Pang's study.

**Figure 3:**
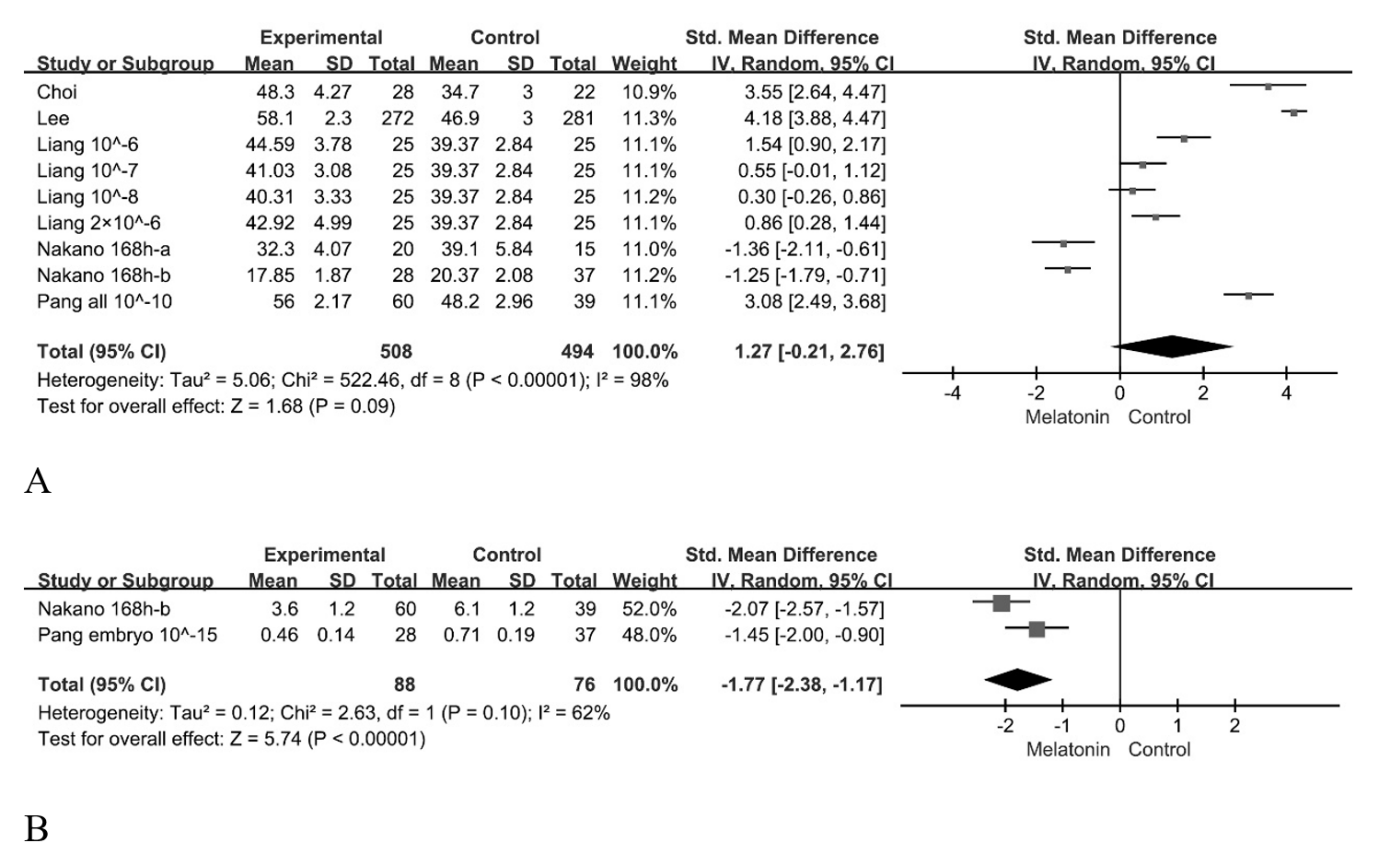
Forest plot of melatonin treatment effects on (A) porcine blastocyst cell number and (B) blastocyst apoptotic cell number. CI = 95%.

Six studies were included in the analysis (Table 2) (Qu et al., 2020a; Lee et al., 2018; Liang et al., 2017; Pang et al., 2013; Nakano et al., 2012; Choi et al., 2008). We found that exogenous melatonin increased the blastocyst rate (95% CI, 1.15~1.42; *p* = 0.58, *I* ^2^ = 0%, fixed effect, Figure 2A). The Funnel plot (Figure 2B) indicated no bias. Melatonin had no effect on cleavage rate (95% CI, 1.00~1.25; *p* = 0.54, *I* ^2^ = 0%, fixed effect, Figure 2C). The number of blastocyst cells was not affected (95% CI, -0.21~2.67; *p* < 0.0001, *I* ^2^ = 98%, random effect, Figure 3A), but the number of apoptotic cells was decreased (95% CI, -2.38–1.17; *p* = 0.1, *I* ^2^ = 62%, random effect, Figure 3B).

**Table 2:** Characteristics of studies included.

**Study**	**ID**	**Year**	**Dose (M)**	**Treat**	**Culture Time (h)**
1	Qu	2020	10^-5 to 10^-11	Embryo	156
2	Lee	2018	10^-9	Embryo	168
3	Liang	2017	10^-6 to 10^-8	Embryo	168
4	Pang	2012	10^-6 to 10^-15	Donor/ Embryo	168
5	Nakano	2011	10^-7	Embryo	144/168
6	Choi	2008	10^-10	Embryo	168

## Discussion

Melatonin is an endogenous indole hormone that is composed of a lipophilic indole heterocycle and a hydrophilic side chain. The side chain contains carbon unsaturated bonds. Therefore, melatonin has hydrophilic, lipophilic, and reductive properties (antioxidative attributes) (Hu et al., 2020; Sanchez-Ajofrin et al., 2020). Melatonin is occurring in prokaryotes and eukaryotes (Hardeland, Fuhrberg, 1996; Hardeland, Poeggeler, 2003; Shibaeva et al., 2018).

### Effects of melatonin on reproductive function

Under natural conditions, the pineal gland controls reproduction by changing the secretion of melatonin according to the length of day and night. As a regulator of the hypothalamic pituitary gonadal axis, melatonin inhibits the expression of some hypothalamic genes, especially neurokinin B, which is a key regulator of GnRH release in long- and short-day animals (Mason et al., 2007). Evidence suggests that neurokinin B may direct seasonal reproduction (Chowdhury et al., 2013). However, it is unclear whether melatonin directly affects GnRH neurons. Additionally, melatonin has direct regulatory effects on secondary reproductive organs (Reiter et al., 2013). A high concentration of melatonin in maternal plasma is necessary for maintaining pregnancy (Latif Khan et al., 2020). Besides scavenging ROS directly, melatonin regulates the expression of antioxidant genes and enzymes (Korkmaz et al., 2012).

Melatonin receptors have been localized in ovarian granulosa cells (Kang et al., 2009), ovarian follicles, and corpora lutea (Soares et al., 2003), as well as in human (Niles et al., 1999) and bovine granulosa cells (Wang et al., 2012). Melatonin can affect ovarian function at the molecular level by downregulating the expression of *CYP11a* and *CYP17* genes and steroid production in porcine thecal cells (Tanavde and Maitra, 2003). The level of melatonin in the ovarian tissue of pre-estrus females is higher than that in normal periods, indicating that the regulation of melatonin concentration is related to the secretion of estrogen. Decreasing the concentration of melatonin in follicular fluid can increase the apoptosis level of granulosa cells and cause follicular atresia (He et al., 2016). Long-term melatonin treatment can delay the aging of the mouse ovary. In addition, studies have shown that the level of melatonin in follicular fluid increases with follicular growth, which may be related to the high level of ROS produced during follicular maturation and the antioxidant properties of melatonin (Futagami et al., 2001).

### Melatonin receptors and the signal transduction process

Melatonin has an important effect on embryo attachment (Dair et al., 2008). Melatonin usually works by inhibiting adenylate cyclase through its membrane receptors MT1 and MT2. During the *in vitro* development of bovine embryos, the MT1 receptor of melatonin was first transcribed on the seventh day of fetal development (Sampaio et al., 2012). Melatonin has a variety of functions in the cell; some functions need receptor participation, while others do not (Reiter et al., 2007). The signal mechanism of melatonin through the membrane receptor is very complex and can vary with different cell types or in different species. Melatonin affects cell physiological functions through membrane receptors (MT1, MT2), nuclear receptors (RZR, ROR), and their interaction with cytoplasmic molecules (e.g., calmodulin) (Dubocovich, 2007; Reiter et al., 2010).

MT1 and MT2 belong to the G-protein coupled receptor family, although their molecular structures, characteristics, and chromosomal localization are different. These two receptors can activate multiple signaling pathways, mainly by inhibiting cAMP formation through sensitive G-protein. Signal transduction cascades associated with the activation of MT1 or MT2 on target cells often result in inhibition of adenosyl cyclase activity (von Gall et al., 2002). The third MT receptor, originally named MT3, is a group of reductases that resist oxidative stress by inhibiting the electrical transfer of quinones (Chen et al., 2013). However, this process has not been identified in the MT3 signaling pathway. The MT1 receptor has been localized in the suprachiasmatic nucleus circadian clock (Dubocovich and Markowska, 2005). It has also been detected in the stromal cells of ovary and testis (Frungieri et al., 2005). However, MT2 receptors are limited in expression. They are mainly expressed in the brain, but they have also been found in the myometrium, granulosa nuclei, and testis. The MT3 receptor has been found in the heart, kidney, brain, oocyte, and ovary (Dubocovich and Markowska, 2005).

Some functions of melatonin require special membrane receptors, while others rely on nuclear receptors. Nuclear receptors are mainly members of RZR and ROR families. Most are considered independent (Smirnov, 2001). Gpr50 of G protein coupled receptors (GPRS) can form dimers with MT1 and MT2. However, this does not change the function of MT2 or antagonize the function of MT1 through heterodimerization (Levoye et al., 2006). There are few studies on the involvement of melatonin in the signal transduction process of embryonic development cells. The main research findings concern the expression of MT1 and MT2 receptors in bovine embryonic development (Sampaio et al., 2012). Most studies suggest that melatonin improves embryonic development ability and reduces the level of apoptosis by regulating ROS. Melatonin significantly promoted the expression of *BMP15*-, *PTX3*-, *HAS2*-, and *EGFR*-related genes of oocyte maturation in sheep (Xiao et al., 2019). The regulation by follistatin on porcine embryonic development is also closely related to the BMP signal transduction pathway (Guo et al., 2018).

### Effect of melatonin on porcine oocytes

The addition of melatonin to the *in vitro* maturation medium or embryo culture medium was beneficial to the parthenogenetic activation of porcine oocytes, and the cleavage rate and blastocyst formation rate were significantly increased (Shi et al., 2009). Melatonin can protect porcine oocytes from the effects of heat stress and improve the maturation rate, cleavage rate, and blastocyst formation rate of porcine oocytes. The results showed that melatonin played a role through its antioxidant function, reduced the formation of ROS in porcine oocytes, and enhanced the production of GSH (Li et al., 2015). Melatonin can promote COC cumulus expansion and subsequent embryo development through the Shh signaling pathway (Lee et al., 2017). Melatonin promotes porcine oocyte maturation by reducing endoplasmic reticulum pressure (Park et al., 2018). Melatonin can promote lipid metabolism of porcine oocytes and provide a necessary energy source for oocyte maturation and subsequent embryonic development (Jin et al., 2017).

### Melatonin and SCNT embryonic development

Many studies have shown that melatonin has a beneficial effect on mammalian gametes and embryos. The damage of free radicals to cell membranes leads to the increase of the sensitivity of lipid peroxidation and changes in protein structure and function, subsequently leading to the destruction of nucleic acids (Leon et al., 2012; Tamura et al., 2012). Melatonin can reduce lipid peroxidation. Melatonin can also improve the stability of sperm DNA (Jang et al., 2009; Fujinoki, 2011). Melatonin also protects oocytes from free radicals during ovulation (Tamura et al., 2008; Reiter et al., 2009). Melatonin improves embryo development *in vitro* (Abecia et al., 2002; Berlinguer et al., 2009). This is consistent with our results. We found that exogenous melatonin can increase the blastocyst rate. However, melatonin did not affect the cleavage rate. This may be due to the short time from the addition of melatonin to cleavage. The cleavage rate was examined within 48 hours after the treatment with melatonin. The electrofusion of rabbit SCNT embryos can induce oxidative stress, disturb epigenetic state, and cause endoplasmic reticulum stress, and melatonin can alleviate these damaging effects (Qu et al., 2020b). Melatonin improves oxidative stress of oocytes and promotes the development of bovine and porcine SCNT embryos *in vitro* (Liang et al., 2017; An et al., 2019).

In the process of *in vitro* production, an increase in the number of apoptotic cells leads to a decrease of embryo viability (Hao et al., 2003). Melatonin downregulates the expression of the apoptosis-promoting genes *Bad* and *Bax*, and upregulates the expression of *Bcl-2*, accompanied by an increase of intracellular glutathione and a decrease of *ROS* (Gao et al., 2012; Mohseni et al., 2012). These changes lead to decreases of blastocyst apoptosis and the improvement of embryo quality and embryo implantation efficiency (Wang et al., 2013). We found that melatonin did not affect the number of blastocyst cells but did decrease the number of apoptotic cells in blastocysts. This effect is crucial for subsequent embryo implantation.

In conclusion, exogenous melatonin can promote oocyte maturation and blastocyst formation. Melatonin reduced ROS levels in porcine oocytes (Choi et al., 2008). In addition, MT1 was expressed in cumulus cells and granulosa cells (Lin et al., 2018). However, according to the literature we have reviewed, there are no reports on the existence of melatonin membrane receptors on porcine oocytes. The effect of melatonin may not only reduce the ROS level in porcine oocytes but also play a role through cell signal transduction pathways. This can also explain why exogenous melatonin does not affect the cleavage rate but can affect the blastocyst rate. Reducing the ROS level through cell signal transduction pathways is a relatively slow process. Our previous experiments also demonstrated that the regulation of signaling pathways in porcine embryo cells resulted in a late gene expression difference (Guo et al., 2018). Therefore, melatonin may be demonstrated to be involved in signal transduction of embryonic cell development in the future.
